# Effects of intravenous inflammasome inhibitor (NuSepin) on suppression of proinflammatory cytokines release induced by cardiopulmonary bypass in swine model: a pilot study

**DOI:** 10.1038/s41598-024-62944-w

**Published:** 2024-06-04

**Authors:** Seung Zhoo Yoon, Jeong Jun Park, Jae Seung Jung, Ji Eon Kim, Seung Hyong Lee, Jeonghoon Lee, Eung Hwi Kim

**Affiliations:** 1 Department of Anesthesiology and Pain Medicine, Korea University College of Medicine, Seoul, Korea; 2 Department of Anesthesiology and Pain Medicine, CHA Bundang Medical Center, CHA University School of Medicine, Seongnam, Korea; 3 Department of Thoracic and Cardiovascular Surgery, Korea University College of Medicine, Seoul, Korea; 4 Institute for Healthcare Innovation, Korea University College of Medicine, Seoul, Korea

**Keywords:** Cardiovascular biology, Drug discovery

## Abstract

The systemic inflammatory response syndrome can occur due to an inflammatory reaction to the release of cytokines, and it has been linked to the circulation of pro- and anti-inflammatory cytokines. The cardiopulmonary bypass (CPB) system is known to activate numerous inflammatory pathways. Applying CPB in large animals for an extended period may be useful as a controlled experimental model for systemic inflammatory responses. The authors hypothesized that 0.2 mg/kg NuSepin^®^ would inhibit CBP-induced proinflammatory cytokine release, and attenuate CPB-induced vasoplegia. CPB was maintained for 2 h in 8 male Yorkshire pigs. Ten ml of saline was administered intravenously to the control group, while the study group received 10 ml of NuSepin^®^ (0.2 mg/kg), before start of CPB. Blood samples were collected at four different time points to evaluating the level of cytokine (TNF-α, IL-1β, IL-6, IL-8) release during and after CBP. All vital signals were recorded as continuous waveforms using the vital recorder^®^. Our study demonstrated that IL-6 increased in both groups during CPB remained unchanged. However, in the Nusepin group, IL-6 levels rapidly decreased when CPB was stopped and the proinflammatory reaction subsided. Furthermore, the dose of norepinephrine required to maintain a mean pressure of 60 mmHg was also lower in the Nusepin group.

## Introduction

The systemic inflammatory response syndrome (SIRS) is a multifaceted reaction that occurs in response to various insults, including infections, traumas, burns, pancreatitis, and other injuries. SIRS is very common in intensive care units (ICU), even in patients who are not infected. Up to 93% of surgical ICU patients meet SIRS criteria, and their mortality rate without infection has been reported to be 7–9%^1^. Furthermore, epidemiological studies have demonstrated that SIRS can serve as a precursor to sepsis^2^. SIRS can arise from an excessive inflammatory reaction to the release of cytokines from injured tissues^3^, and it has been associated with the circulation of pro- and anti-inflammatory cytokines^4^. Although it is not yet proven, high levels of proinflammatory cytokines have been strongly linked to adverse outcomes following surgery. While a direct causal relationship has not been established, there is a strong association between elevated proinflammatory cytokines and unfavorable outcomes following surgery. Among these cytokines, IL-6 has been proposed as a potential marker whose levels are directly correlated with the risk of postoperative complications^5–7^.

Until the 1950s when the cardiopulmonary bypass (CPB) system was developed, cardiovascular surgery had a high mortality and morbidity rate^8,9^. However, the use of CPB is associated with the activation of various inflammatory pathways involving cellular elements such as red blood cells, platelets, and white blood cells, as well as soluble proteins. While complement activation typically occurs during surgical procedures, the response is more pronounced in cardiac surgeries using CPB. The extent of complement activation is directly proportional to the duration of CPB^10^. Cardiovascular surgery with CPB often triggers a systemic inflammatory response syndrome, which significantly affecting the postoperative mortality and morbidity^11–13^. Critically ill patients have limited capacity to counterbalance inflammatory responses, making them more susceptible to endothelial damage and capillary leak syndrome. Although all organs can experience increased tissue edema, the lungs, brain, kidneys, and myocardium are especially vulnerable. Therefore, post-CPB syndrome can manifest as noncardiogenic pulmonary edema, myocardial dysfunction, severe vasoplegia, hemodynamic instability, renal dysfunction, and in severe cases, multiorgan failure^14^. As a result, the application of long-term CPB in large animals can be beneficial as a controlled experimental model for studying systemic inflammatory responses.

NuSepin^®^ is a novel anti-inflammatory material based on taurodeoxycholate (TDCA). TDCA is a GPCR19 agonist^15^ that suppresses NLRP3 inflammasome activation^16^. TDCA effectively suppresses the production of inflammatory cytokines, including TNF-α, MCP-1, IL-6, and IL-1β, in LPS-induced sepsis models in mice. TDCA also increases the number of myeloid-derived suppressor cells (MDSC) and controls alternative splicing, chromatin silencing, and translation of the immune proteome of MDSC, which increases the anti-inflammatory potency of MDSCs^16^. In phase 1 clinical trial, healthy volunteers received 0.1 mg/kg, 0.2 mg/kg, 0.4 mg/kg, 0.8 mg/kg, 1.6 mg/kg NuSepin^®^, or placebo. Among 39 subjects who received NuSepin^®^, there was no serious adverse events. All adverse events by NuSepin^®^ treatment resolved spontaneously without any treatment. In phase 2 clinical trial, COVID-19 pneumonia patients who received 0.2 mg/kg NuSepin^®^ showed 1.34-fold higher recovery rate ration and 50% decreased serum pro-inflammatory cytokine level (CRP, IL-6, TNF-α, IL-8) compared to placebo group^17^.

Therefore, the authors hypothesized that 0.2 mg/kg NuSepin^®^, administered intravenously immediately after anesthesia induction, 1) would inhibit CBP-induced proinflammatory cytokine release, and that 2) inhibition of proinflammatory cytokine release, in turn, would attenuate CPB-induced vasoplegia. Therefore, the primary end-point of the study is to evaluate the association between NuSepin^®^ administration and plasma pro-inflammatory cytokine levels during and after CPB. The secondary end-point of the study is to determine whether NuSepin^®^ can reduce the need for vasoconstrictive agents, specifically norepinephrine, after CPB.

## Results

Eight male Yorkshire pigs, aged 11–12 weeks and weighing 40.5 ± 0.76 kg, were utilized in this study to minimize hormonal variations. There were no significant differences in characteristic data, pre-CPB time, CPB time, baseline hemodynamic values, or baseline cytokine levels between the groups (Table 1). Baseline hemodynamic values were the average of 10 min of hemodynamic vital data measurements after the completion of arteriovenous cannulation.

**Table 1:** Pig characteristics, baseline hemodynamic values, baseline level of cytokines.

	Nusepin Group (n = 4)	Control Group (n = 4)	*p* value
Age (weeks)	11.5 ± 0.4	11.6 ± 0.3	0.69
Weight (kg)	40.6 ± 0.4	40.7 ± 0.4	0.89
PreCPB time (min)	80.5 ± 5.3	81.8 ± 4.3	0.69
CPB time (min)	120 ± 0	120 ± 0	1
Baseline hemodynamic values			
SBP (mmHg)	99 ± 6	105 ± 5	0.53
MBP (mmHg)	72 ± 5	76 ± 5	0.53
DBP (mmHg)	58 ± 5	61 ± 5	0.80
HR (bpm)	95 ± 15	100 ± 20	0.64
CO (L/min)	5.6 ± 0.5	6.5 ± 0.3	0.11
SV (ml)	52.8 ± 8.9	61.7 ± 8.7	0.63
CVP (mmHg)	15.8 ± 4.5	13.4 ± 1.9	0.63
SVR (mmHg*sec/ml)	768.5 ± 68.4	830.7 ± 46.6	0.63
Baseline level of cytokines			
TNF-alpha (pg/ml)	94.1 ± 15.6	102.2 ± 20.3	0.34
IL-1β (pg/ml)	122.5 ± 9.6	133.1 ± 9.7	0.49
IL-6 (pg/ml)	75.8 ± 3.4	86.4 ± 11.9	0.34
IL-8 (pg/ml)	21.0 ± 8.1	23.8 ± 11.4	0.34

Baseline hemodynamic values are the average of 10 min of hemodynamic vital data measurements after the completion of arteriovenous cannulation.

PreCPB time: Time from immediately after anesthesia to initiation of cardiopulmonary bypass (CPB), SBP: Systolic blood pressure, MBP: Mean blood pressure, DBP: Diastolic blood pressure, HR: Heart rate, CO: Cardiac output, SV: Stroke volume, CVP: Central venous pressure, SVR: Systemic vascular resistance.

**Figure 1:**
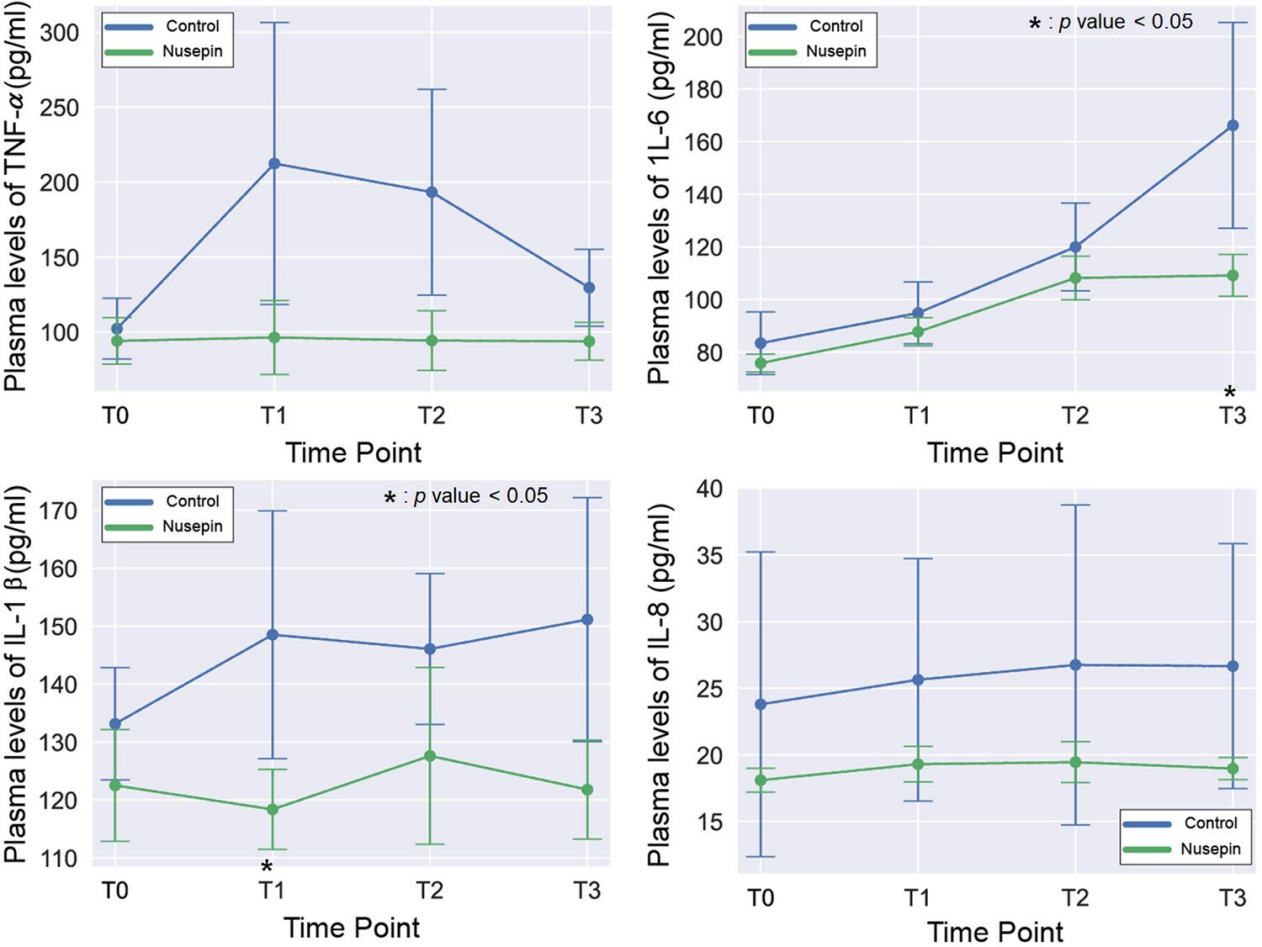
Changes of plasma concentration levels of cytokines. Overall, the plasma cytokine concentration levels tended to be lower in the NuSepin^®^-treated group. Except for T1(lower in Nusepin group), the IL-1β showed no difference between the two groups. TNF-α showed no statistical difference between the two groups at each time point. However, plasma concentration of TNF-α at T1 and 2 was 30% lower in Nusepin group (*p* = 0.057). Except for T3 (lower in Nusepin group), IL-6 showed no difference between the two groups. IL-8 did not show a difference between the two groups. (T0: pre-CPB/T1: CPB 60 min/T2: CPB 120 min/T3: Post-CPB 120 min).

Figure 1 show changes of plasma concentration levels of cytokines and Table 2 show magnitude of changes in plasma cytokine levels compared to baseline: pre-CPB(T0), CPB 60 min(T1), CPB 120 min(T2), and post-CPB 120 min(T3). Overall, the plasma cytokine concentration levels tended to be lower in the Nusepin group. The IL-1β showed no difference between the two groups except for T1(lower in the Nusepin group). TNF-α showed no statistical difference between the two groups at each time point. However, plasma concentration of TNF-α at T1 and 2 was 30% lower in Nusepin group (*p* = 0.057). Il-6 showed no difference between the two groups except for T3 (lower in the Nusepin group). IL-8 did not show a sdifference between the two groups.

**Table 2:** Changes in plasma cytokine concentrations at each time point.

		Nusepin group [measured value]	Control group [measured value]	*p* value
TNF-α	T0	100.00 [94.1 ± 15.5]	100.00 [102.2 ± 20.3]	
	T1	103.36 ± 23.09 [96.4 ± 24.6]	224.89 ± 149.19 [212.4 ± 94.1]	0.114
	T2	100.49 ± 12.94 [94.3 ± 19.9]	198.53 ± 99.35 [193.3 ± 68.6]	0.114
	T3	100.74 ± 11.25 [93.9 ± 12.6]	132.12 ± 46.87 [129.6 ± 25.6]	0.343
IL-1β	T0	100.00 [122.5 ± 9.6]	100.00 [133.1 ± 9.7]	
	T1	96.88 ± 6.10 [118.4 ± 6.9]	112.11 ± 18.78 [148.5 ± 21.4]	0.200
	T2	104.61 ± 14.57 [127.6 ± 15.3]	110.02 ± 11.26 [146.1 ± 13.0]	0.486
	T3	99.53 ± 4.17 [121.8 ± 8.6]	113.50 ± 13.83 [151.1 ± 21.1]	0.057
IL-6	T0	100.00 [75.8 ± 3.4]	100.00 [83.4 ± 11.9]	
	T1	115.86 ± 8.73 [87.7 ± 5.4]	114.21 ± 8.41 [94.9 ± 11.8]	0.886
	T2	143.16 ± 15.65 [108.2 ± 8.3]	144.65 ± 17.09 [120.0 ± 16.7]	0.886
	T3	144.00 ± 9.03 [109.2 ± 7.9]	201.40 ± 50.70 [166.2 ± 39.1]	0.057
IL-8	T0	100.00 [18.1 ± 0.9]	100.00 [23.9 ± 11.5]	
	T1	106.60 ± 4.34 [19.3 ± 1.3]	117.02 ± 27.22 [25.6 ± 9.1]	0.686
	T2	107.39 ± 5.29 [19.5 ± 1.5]	116.76 ± 16.78 [26.8 ± 12.0]	0.688
	T3	104.48 ± 0.96 [19.0 ± 0.8]	125.15 ± 41.74 [26.7 ± 9.2]	0.343

Data are expressed as % change of mean value from T0 ± standard deviation.

**Figure 2:**
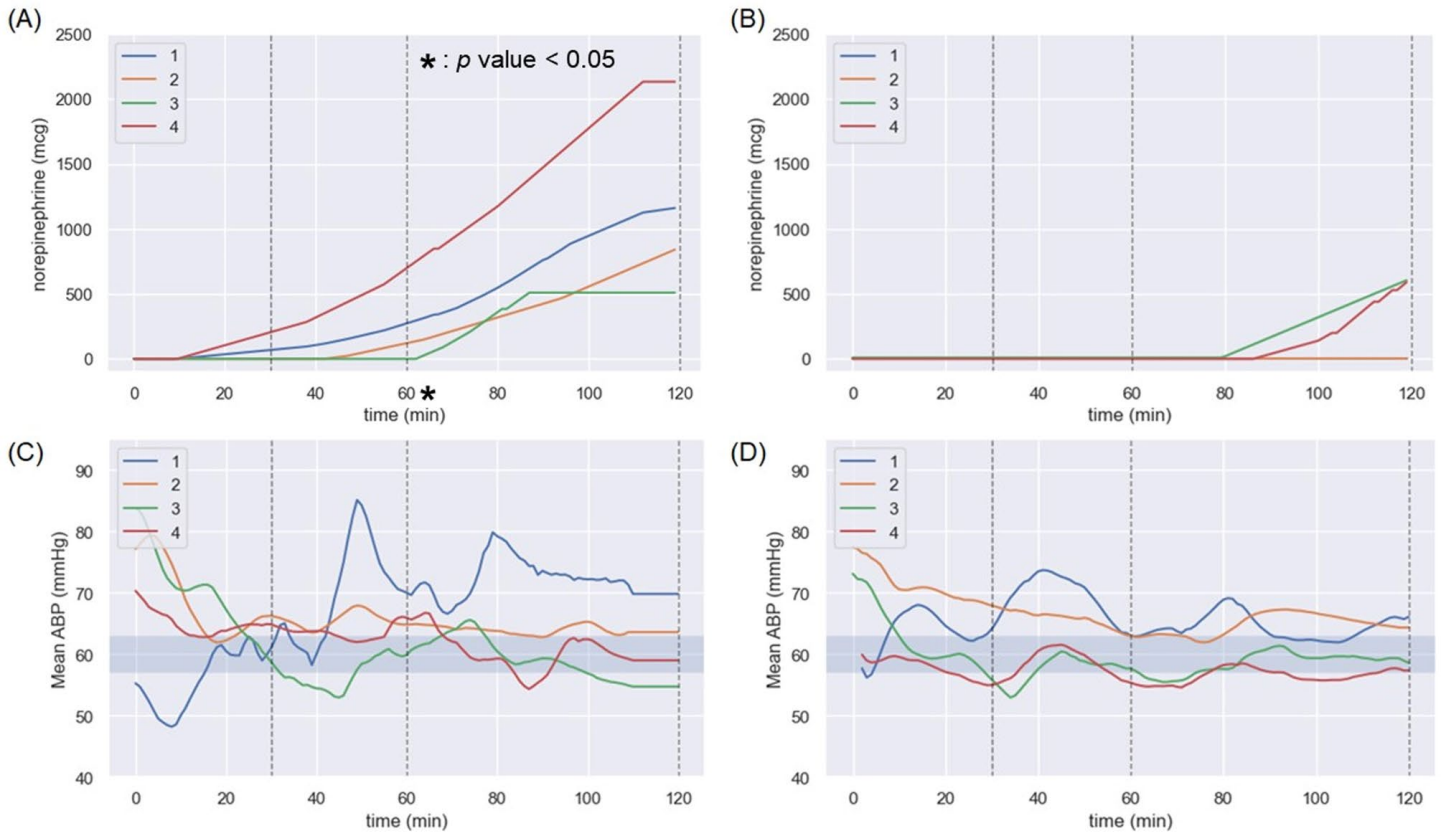
Total amount of the norepinephrine use over time and MBP of the experimental animal. (**A**) Total amount of the norepinephrine use of control group. (**B**) Total amount of the norepinephrine use of Nusepin group. (**C**) Mean Blood Pressure of control group. (**D**) Mean Blood Pressure of Nusepin group. After 30 min of weaning from CPB, norepinephrine was started to maintain mean pressure above 60 mmHg in all Control groups and in 2 Nusepin group. Thereafter, continuous infusion of norepinephrine was continued for these 6 animals until the end of the experiment. The total amount of norepinephrine administered was significantly higher in the control group after 60 min of weaning from CPB. The mean of the total amount of norepinephrine used during post-CPB 120 min was 273 mcg and 2.5 mcg in the control and Nusepin group, respectively (*p* = 0.048).

Figure 2 illustrate the total amount of norepinephrine use over time. After 30 min of weaning from CPB, norepinephrine was started to maintain mean pressure above 60 mmHg in all of the control group and in 2 of the Nusepin group. Thereafter, continuous infusion of norepinephrine was continued for these 6 animals until the end of the experiment. The total amount of norepinephrine administered was significantly higher in the control group after 60 min of weaning from CPB.

**Figure 3:**
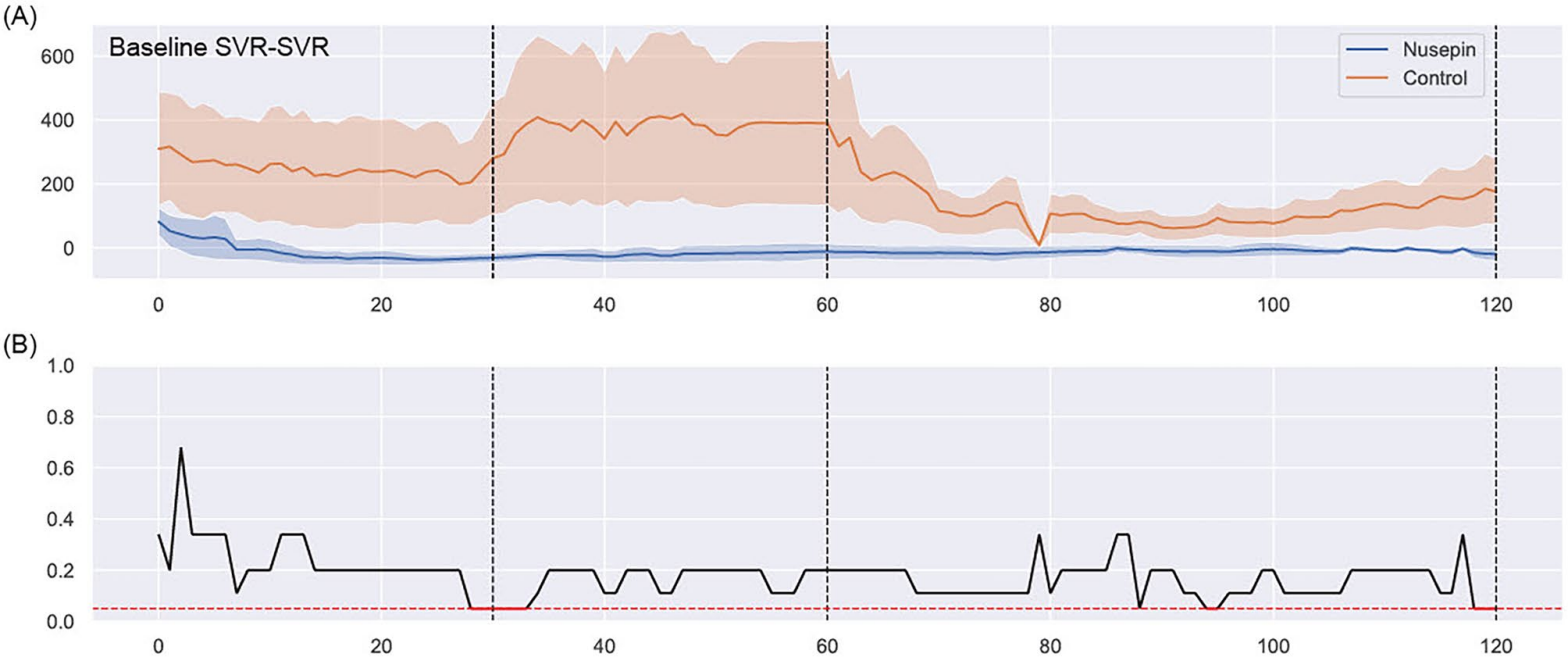
Continuous Systemic Vascular Resistance comparison. (**A**) Subtraction of SVR from baseline SVR in the Nusepin group and the control group. The vertical line is the value of baseline SVR-SVR. The plotted line on the graph represents the mean value, while the data points are depicted with markers indicating the standard deviation. (**B**) A statistical analysis of the difference between the two groups. Red marks the interval where the difference between the two groups was statistically significant (*p* value < 0.05) when comparing SVR at consecutive time points. It shows that the Nusepin group is maintained without significant difference from the baseline SVR even after CPB weaning. On the other hand, in the control group, the difference from the baseline SVR was large and fluctuating until 60 min. It showed a relatively stable pattern after 80 min of CPB weaning. Immediately after weaning from CPB, in addition, baseline SVR -SVR was measured lower in the Nusepin group.

Figure 3 shows baseline SVR—measured SVR after weaning from CPB. It shows that the SVR of Nusepin group is maintained uneventfully without significant difference from the baseline SVR even after CPB weaning. On the other hand, in the control group, the difference from the baseline SVR was large and fluctuating until 60 min of CPB weaning. It showed a relatively stable pattern after 80 min of CPB weaning. Immediately after weaning from CPB, in addition, baseline SVR–SVR was measured statistically lower in the Nusepin group.

**Figure 4:**
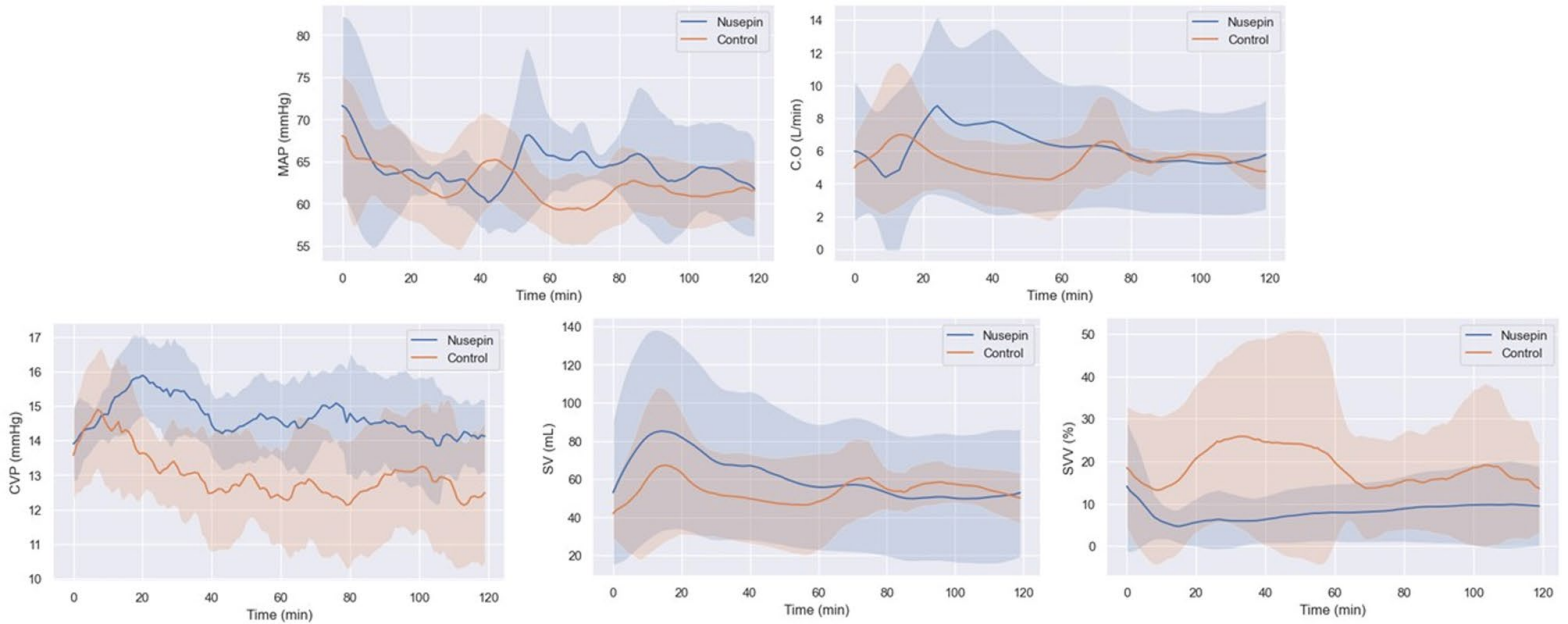
Comparison of continuous hemodynamic data during post-CPB 120 min. The continuous hemodynamic during post-CPB 120 min was not statistically significant between the two groups. However, CVP and SV tended to remain relatively higher in the Nusepin group compared to the control group. In addition, SVV tended to be lower in the Nusepin group. This shows that the Nusepin group ether tends to be stable in terms of hemodynamic stability.

Figure 4 shows no differences were found between the groups in any hemodynamic parameters, including blood pressure, cardiac output, stroke volume, stroke volume variation, and central venous pressure.

## Discussion

Our study demonstrated that IL-6 increased in both groups during CPB. However, in the Nusepin group, IL-6 levels rapidly decreased when CPB was stopped and the proinflammatory reaction subsided. Furthermore, the dose of norepinephrine required to maintain a mean pressure of 60 mmHg was also lower in the Nusepin group.

IL-1β and TNF-α, in particular, are known to activate immune cells and promote the release of other inflammatory mediators, leading to tissue damage and dysfunction. IL-6 also contributes to the inflammatory response, but it also has anti-inflammatory effects and is involved in tissue repair and regeneration^18,19^. When a substance that suppresses the inflammatory response, such as NuSepin^®^, is administered, it can reduce the secretion of these proinflammatory cytokines. This can occur through various mechanisms, including the inhibition of signaling pathways that lead to their production or the suppression of the immune cells responsible for their release. IL-1β secretion decreases in response to the suppression of the inflammasome pathway. The inflammasome is a protein complex that is activated in response to various stimuli, including pathogenic microorganisms and cellular damage^20^. It promotes the secretion of IL-1β by immune cells, and its suppression can lead to decreased IL-1β production. TNF-α secretion decreases due to the inhibition of its transcription and translation. NuSepin^®^, for example, can inhibit the expression of TNF-α by suppressing the activation of transcription factors that promote its production. It can also directly inhibit the translation of TNF-α mRNA into protein. IL-6 decreases due to the suppression of immune cell activation and cytokine signaling pathways. NuSepin^®^, for example, can suppress the activation of immune cells such as monocytes and macrophages, which are major sources of IL-6 production. It can also inhibit the signaling pathways involved in IL-6 production and secretion, such as the JAK/STAT pathway^16,21^. Therefore, we may suggest that overally suppression of proinflammatory cytokines such as IL-1β, TNF-α, and IL-6 by substances like NuSepin^®^ can help to reduce the severity of the inflammatory response and mitigate tissue damage. In the present study, no significant differences in IL-8 levels were observed between the NuSepin and control groups. Considering IL-8 is recognized as a pro-inflammatory cytokine associated with tissue damage in regions affected by infection, our results may be contrasts with previous studies. However, it's worth noting that our experiment was conducted within a non-infectious systemic inflammatory response syndrome (SIRS) context, specifically tailored to emulate the clinical setting of heart valve surgery. The absence of infection in our study may offer an explanation for the disparate results compared to previous experiments. This discrepancy underscores the importance of considering the specific experimental conditions and contexts when interpreting cytokine responses and their implications.

Several factors seemingly contribute to the systemic inflammatory response, including the interaction of blood with the surface of the cardiopulmonary bypass (CPB) device, the trauma from the surgical procedure, endotoxemia, blood loss, and ischemic reperfusion injury^22^. In the context of blood contact with CPB circuits, it has been observed that while the classical complement pathway can be activated through C1 activation by FXIIa^23^, the alternate complement pathways appear to exert a dominant influence in the production of anaphylatoxins such as C3a and C5a, as well as the formation of the terminal membrane attack complex (C5b-9)^24^. The activation of neutrophils, triggered by anaphylatoxins and kallikreins, results in the release of lytic enzymes such as elastase and oxygen free radicals. Both of these factors can instigate tissue damage and dysfunction of organs^25^. During CPB, both complement activation and neutrophil activation contribute to the increased levels of cytokines^26^. Interleukin (IL)-1, IL-6, IL-8, and tumor necrosis factor (TNF) are among the proinflammatory cytokines that exhibit elevated levels in response to tissue injury and endotoxic challenge. These cytokines play a crucial role in the pathogenesis of post-CPB inflammatory syndrome and sepsis^27^. Among these cytokines, IL-6 has been proposed as a potential marker directly associated with the risk of postoperative complications^28^.

In 2003, the mortality rates following isolated coronary artery bypass grafting (CABG) were reported to be 2.4%, while after aortic valve replacement it was 3.5%, and after mitral valve replacement it was 5.5%. Furthermore, the occurrence of major complications in adult cardiac surgery is also significant. Myocardial infarction is observed in 2–10% of patients, with the incidence varying depending on the definition used. Stroke occurs in approximately 2–3% of cases, renal failure in 3% of cases, and pulmonary complications necessitating prolonged mechanical ventilation in the intensive care unit occur in 3–4% of patients^29^. In recent decades, various protective strategies have been implemented to enhance safety during CPB procedures. These strategies include the utilization of bubble traps and screen filters to decrease the likelihood of embolization, the implementation of lung-protective mechanical ventilation techniques to prevent respiratory distress syndromes, advancements in pH, temperature, and fluid management, and the adoption of pulsatile flow (which has demonstrated improvements in renal function and regional oxygen saturation of the brain).

Additionally, pharmacological approaches have been explored to minimize organ damage during CPB procedures^30–32^. Pharmacological strategies aimed at suppressing the systemic inflammatory response to surgery have largely evolved from the utilization of agents already employed for hemostasis and coagulation management, such as aprotinin and heparin-coated circuits. The development of dedicated anti-inflammatory agents targeting the inflammatory response has been limited, and those that have been developed often exhibit a narrow therapeutic focus. This narrow targeting may hinder their ability to effectively mitigate the multi-system etiology of the systemic inflammatory response. Furthermore, in order to address the safety concerns associated with the use of anti-fibrinolytics in cardiac surgery, it is imperative that future clinical trials are adequately supported to detect well-defined adverse events such as stroke, myocardial injury, and acute renal failure requiring dialysis^33^.

Vasoplegia, a condition characterized by severe hypotension and vasodilation, is a common complication of cardiac surgery with CPB. The pathophysiology of vasoplegia involves multiple mechanisms, including the activation of the inflammatory response, oxidative stress, and the depletion of catecholamines^10^. In this study, the administration of NuSepin^®^, a substance that suppresses the inflammatory response, was found to reduce the levels of proinflammatory cytokines and the amount of norepinephrine required to maintain mean arterial pressure above 60 mmHg. This suggests that the suppression of the inflammatory response by NuSepin^®^ may have contributed to the decrease in vasoplegia observed in the Nusepin group. Furthermore, the maintenance of SVR in the Nusepin group, as demonstrated by the stable pattern of SVR after weaning from CPB, may have also played a role in reducing vasoplegia. SVR is a key determinant of blood pressure, and the maintenance of SVR in the Nusepin group suggests that the vasodilatory response, which is a characteristic feature of vasoplegia, was attenuated. Overall, the reduction in proinflammatory cytokines, the decreased amount of norepinephrine required to maintain mean arterial pressure, and the maintenance of SVR all suggest that the administration of NuSepin^®^ may have contributed to the decrease in vasoplegia observed in this study.

Reducing vasoplegia can have positive effects on postoperative outcomes in several ways. First, by maintaining vascular tone and blood pressure, it can improve organ perfusion and oxygenation, which can reduce the incidence of postoperative organ dysfunction and failure. Second, reducing vasoplegia can decrease the need for vasopressor support, such as norepinephrine, which is associated with adverse effects, such as myocardial ischemia and arrhythmias. Third, it can decrease the incidence of postoperative complications and prolonged hospital stay, which can ultimately lead to better patient outcomes and reduced healthcare costs. In the context of the current study, the reduced vasoplegia observed in the Nusepin group may have contributed to the lower levels of proinflammatory cytokines. This, in turn, may have led to better postoperative outcomes, such as faster recovery and reduced incidence of complications. However, it is important to note that this study only investigated short-term effects, and further studies are needed to assess the long-term effects of NuSepin^®^ on postoperative outcomes.

## Conclusion

This study demonstrated that the proinflammatory cytokine release remained unchanged during CPB. However, in the Nusepin group, proinflammatory cytokine levels tend to be stabilized when CPB was stopped. Furthermore, the dose of norepinephrine required to maintain MBP was also lower in the Nusepin group.

## Limitations

This study is an animal test of the effects of a new drug and has several limitations.

First, as this study represents novel experimentation, the authors conducted it as a pilot study with approval from the Korea University Animal Experimentation Ethics Committee for a total of 8 animals. This number was determined as the minimum required, adhering to the 3Rs (Replacement, Reduction, Refinement) principle. Due to the limited sample size, the data did not exhibit statistically normal distribution overall. However, given the small sample size, statistical significance might not always reflect true biological differences. Thus, while some results showed significant differences between the two groups, cautious interpretation is warranted. Nevertheless, the authors were able to discern trends that they believe will be valuable for future investigations.

Second, the authors used isoflurane as a method of maintaining anesthesia. Isoflurane is known to have an anti-inflammatory effect^34^. Although it was used equally in both groups, it is possible that the effect of the experimental drug was reduced by the anti-inflammatory effect of isoflurane itself. Third, the authors chose to use only male pigs for their analysis to reduce hormonal variation. However, this introduction of sex bias leads to results that may be less applicable across sexes.

Finally, based on the observations that IL-1β levels did not significantly differ between the groups for most time points, the characterization of NuSepin^®^ as an effective inflammasome inhibitor is called into question. However, drawing conclusive interpretations from the current study results would be unwarranted. Therefore, this discrepancy between the expected function of NuSepin^®^ and the study findings necessitates further clarification and investigation.

## Methods

### Ethics statement

This animal study received approval from the Korea University Institutional Animal Care and Use Committee (KUIACUC, Approval: KOREA-2022-0024-C2) and conducted in accordance with the relevant animal ethics guidelines. All experimental procedures, data acquisition, interpretation, and analysis pertaining to live animal experiments were conducted in accordance with the Animal Research: Reporting of In Vivo Experiments (ARRIVE) guidelines.

### Animal

Eight male Yorkshire pigs weighing 40.5 ± 0.76 kg and aged 11–12 weeks were utilized for the study. The pigs were sourced from GP Farms, Orient Bio, South Korea as 'bacon pigs' and acclimatized to the Animal Facility at Korea University for 2 weeks prior to the study. The animals were housed in individual raised pens (2 × 2 m), fed a maintenance diet (Purina Feeds Ltd. pig fodder), and provided free access to tap water in a temperature-controlled environment (23 ± 2.3 °C) under a 12:12 h light/dark cycle. The pigs were fasted for 24 h before the surgery.

### Anesthesia

The anesthetic agents used included Rompun^®^ (xylazine, 1 mg/kg, Dechra; USA) and Zoletil^®^ (zolazepam/tiletamine, 7 mg/kg, Virbac; New Zealand), which were administered intramuscularly. The animals were intubated with a standard cuffed endotracheal tube (Portex^®^, 6 mm internal diameter, Smith-Medical; USA), and the cuff was inflated with 15 ml of air. The correct position of the endotracheal tube was confirmed by chest expansion, bilateral chest auscultation, absence of air leakage around the cuff, and end-tidal CO2 detection via capnography. Anesthesia was maintained using isoflurane at an end-tidal concentration of 1.5%. Fresh gas flow was maintained at 4 L/min with FiO2 of 0.5. Tidal volume and respiratory rate were set at 8 ml/kg and 15 breaths/min, respectively. The animals were placed in a supine position with neck extended on surgical tables and monitored using standard anesthetic monitors, including electrocardiogram, pulse oximeter, and non-invasive blood pressure. A 3-lm central venous catheter (Arrowg + ard Blue Plus^®^, Teleflex; North Carolina, USA) was inserted into the femoral vein to monitor central venous pressure (CVP), and Ringer's lactate solution was administered at 5 ml kg^−1^ h^−1^ during anesthesia. An arterial catheter was inserted into the femoral artery to monitor arterial blood pressure (ABP). All hemodynamic data, including ABP, CVP, stroke volume (SV), stroke volume variance (SVV), and cardiac output (CO), were monitored using the EV1000^®^ with FloTrac sensor (Edwards Lifesciences; Irvine, USA). All drugs were injected using an Agilia^®^ infusion pump (Fresenius Kabi; Bad Homburg, Germany). All vital signals observed using the anesthetic monitoring device were recorded as continuous waveforms through the vital recorder^®^ (VitalDB; Seoul, Korea).

When weaning from the cardiopulmonary bypass (CPB), a continuous infusion of norepinephrine was used to maintain a mean blood pressure above 60 mmHg, while normal saline was used to keep the stroke volume variation (SVV) below 15.

### Study drug administration

The study drugs were prepared by an independent researcher who did not participate in experiment. All the cardiac surgeons, anesthetic providers, and investigators who collected the data were blinded to the group assignment. After anesthesia induction and the placement of an intravenous line, pigs were randomly assigned to either a control group or a study group using a coin toss. The control group received 10 ml of saline intravenously, while the study group received 10 ml of NuSepin^®^ (0.2 mg/kg) intravenously. After injection, the pigs were prepared for surgery, and cannulations were performed to start CPB.

### Cardiopulmonary bypass

In general, the CPB time for heart valve surgery by experienced operators is less than 2 h. Considering the clinical situation, the authors set the CPB time to 2 h**.** Before CPB initiation, the tidal volume was adjusted to achieve norm ventilation with oxygen in air (FiO2 0.5) and was controlled by blood gas analysis to maintain normal arterial carbon dioxide tension. The study was performed using standard CPB technique (2.4 l/min/m^2^) with moderate hypothermia (nasopharyngeal temperature 32–34 °C), roller pumps and the administration of heparin (300 IU/kg). The activated clotting time was maintained above 400 s. The circuit was primed with Ringer's lactated solution, albumin, and mannitol. The cannulation strategy used a standard Bicaval technique venous drainage (32 and 40 Fr, Medtronics, Minenesota, USA) and return to the distal ascending aorta (16 Fr, Medtronics, Minenesota, USA). After weaning from the CPB (rectal temperature 36.5–37 °C), anticoagulation activity was reversed with protamine sulfate given at a ratio of 1 mg: 100 IU of heparin. The CPB weaning protocol is as follows: partially clamp the venous line and gradually increase the preload on the venous drain. Observe the waveform of the arterial line with ejection as the preload increases and gradually reduce the venous drain to maintain static arterial pressure. CPB was stopped when pump flow was less than 10%.

### Blood sample

Blood samples were collected immediately after anesthesia induction (time point 0, T0), 1 h after CPB initiation (time point 1, T1), 2 h after CPB initiation (time point 2, T2), and 2 h after weaning from the CPB (time point 3, T3). Considering that most cardiac surgeries are completed within 2 h after CPB, blood samples were collected up to 2 h after CPB termination, and after the completion of sampling, the pigs were euthanized using potassium. All samples were taken from the cannulated femoral artery, and anticoagulated with ethylene diamine tetra-acetic acid, immediately cooled to 4 °C, centrifuged within 10 min, and stored at −80 °C until assay. TNF-α, IL-1β, IL-6 and IL-8 serum levels were measured using commercially available enzyme-linked immunosorbent assays (ELISA) to evaluate the level of proinflammatory cytokine release.

### Statistical analysis

To compare categorical data, the chi-square test was used. For nonparametric data, the Mann–Whitney test was employed. All values are expressed as mean ± standard deviation (SD) and were compared based on the distribution of variables. The Mann–Whitney U test and Kruskal–Wallis test were used to compare levels of TNF-α and IL-1b, 6,8. Norepinephrine usage and SVR between the two groups was compared by the sum of the integrals of the lower part of the usage curve. Statistical analyses were performed using IBM SPSS Statistics 22.0 (IBM Corp. Armonk, NY, USA). Hemodynamic data were analyzed using the general linear mixed model followed by Tukey–Kramer correction. A *p* value of < 0.05 was considered statistically significant.

### Supplementary Information

Supplementary Information.

## Data Availability

The datasets used and/or analyzed during the current study available from the corresponding author on reasonable request.
