# Supplementary material for: Effects of intravenous inflammasome inhibitor (NuSepin) on suppression of proinflammatory cytokines release induced by cardiopulmonary bypass in swine model: a pilot study
Source: Sci Rep. 2024 Jun 4;14:12797. doi: 10.1038/s41598-024-62944-w (PMC11150435; doi:10.1038/s41598-024-62944-w)
Supplement: Supplementary file 1 — Supplementary Information. [file 41598_2024_62944_MOESM1_ESM.docx]

**Effects of intravenous inflammasome inhibitor (NuSepin) on suppression of proinflammatory cytokines release induced by cardiopulmonary bypass in swine model: A Pilot Study**

Seung Zhoo Yoon^1,*,^ ^+^, Jeong Jun Park^2,^ ^+^, Jae Seung Jung^3^, Ji Eon Kim^3^, Seung Hyong Lee^3^, Jeonghoon Lee^1^ and Eung Hwi Kim^4^

^1^Department of Anesthesiology and Pain Medicine, Korea University College of Medicine, Seoul, Korea

^2^Department of Anesthesiology and Pain Medicine, CHA Bundang Medical Center, CHA University School of Medicine, Seongnam, Korea

^3^Department of Thoracic and Cardiovascular Surgery, Korea University College of Medicine, Seoul, Korea

^4^Institute for Healthcare Innovation, Korea University College of Medicine, Seoul, Korea

| **Page** | **Table of Content** |
| --- | --- |
| P1 | Title page |
| P2 | Table S1. |

**Table S1.** Raw data of plasma cytokine levels at each time points

|  |  | Nusepin | Control |  |  | Nusepin | Control |
| --- | --- | --- | --- | --- | --- | --- | --- |
| TNF-α | T0 | 97.38 | 75.11 | IL-6 | T0 | 80.15 | 100.112 |
|  |  | 109.22 | 114.49 |  |  | 75.357 | 79.7225 |
|  |  | 97.38 | 98.73 |  |  | 71.902 | 72.1275 |
|  |  | 72.31 | 120.55 |  |  | 75.87 | 81.5975 |
|  | T1 | 90.15 | 332.41 |  | T1 | 83.43 | 111.971 |
|  |  | 131.60 | 208.20 |  |  | 94.068 | 85.0725 |
|  |  | 74.00 | 102.43 |  |  | 83.067 | 91.07 |
|  |  | 89.93 | 206.62 |  |  | 90.265 | 91.39 |
|  | T2 | 89.92 | 250.33 |  | T2 | 96.092 | 142.775 |
|  |  | 123.53 | 223.41 |  |  | 111.716 | 109.7225 |
|  |  | 84.43 | 94.17 |  |  | 110.184 | 121.8625 |
|  |  | 79.40 | 205.31 |  |  | 114.77 | 105.5925 |
|  | T3 | 94.76 | 150.67 |  | T3 | 110.355 | 156.509 |
|  |  | 111.29 | 126.48 |  |  | 106.42 | 147.56 |
|  |  | 86.06 | 94.56 |  |  | 100.46 | 137.095 |
|  |  | 83.43 | 146.63 |  |  | 119.3925 | 223.6675 |
| IL-1β | T0 | 131.527 | 125.37 | IL-8 | T0 | 18.477 | 30.068 |
|  |  | 129.324 | 147.18 |  |  | 17.57 | 34.828 |
|  |  | 110.989 | 128.68 |  |  | 19.173 | 21.539 |
|  |  | 118.205 | 131.29 |  |  | 17.189 | 8.735 |
|  | T1 | 128.526 | 132.63 |  | T1 | 20.88 | 33.405 |
|  |  | 115.98 | 134.69 |  |  | 18.536 | 31.878 |
|  |  | 115.951 | 147.73 |  |  | 19.888 | 23.667 |
|  |  | 113.039 | 178.99 |  |  | 17.905 | 13.59 |
|  | T2 | 135.478 | 132.71 |  | T2 | 21.296 | 33.366 |
|  |  | 112.67 | 144.41 |  |  | 18.235 | 39.31 |
|  |  | 117.21 | 143.15 |  |  | 20.133 | 22.023 |
|  |  | 145.05 | 163.93 |  |  | 18.136 | 12.313 |
|  | T3 | 133.898 | 127.89 |  | T3 | 19.512 | 29.477 |
|  |  | 120.95 | 160.56 |  |  | 18.398 | 37.867 |
|  |  | 114.098 | 140.62 |  |  | 19.851 | 22.933 |
|  |  | 118.208 | 175.43 |  |  | 18.136 | 16.368 |
